# Enhancing cropping intensity and system productivity through early-bulking potato genotypes in potato-based cropping systems

**DOI:** 10.1371/journal.pone.0344679

**Published:** 2026-03-17

**Authors:** Md. Mostahed Hossain, A. K. M. Mominul Islam, Jerina Tasnim, Md. Abdur Rahman Sarkar, Naresh Chandra Deb Barma, Ahmed Khairul Hasan

**Affiliations:** 1 Regional Agricultural Research Station, Bangladesh Agricultural Research Institute, Burirhat, Rangpur, Bangladesh; 2 Department of Agronomy, Bangladesh Agricultural University, Mymensingh, Bangladesh; 3 Department of Agrometeorology, Bangladesh Agricultural University, Mymensingh, Bangladesh; 4 Krishi Gobeshona Foundation (KGF), BARC Complex, Farmgate, Dhaka, Bangladesh; University of Florida Tropical Research and Education Center, UNITED STATES OF AMERICA

## Abstract

Increasing cropping intensity is a key strategy for enhancing food production in regions facing shrinking arable land and rising population pressure. Early-bulking potato genotypes offer opportunities to redesign potato-based cropping systems by creating temporal space for additional crops. This study evaluated the agronomic performance, system productivity, economic returns, and land use efficiency of early-bulking potato genotypes (7 *Alu* and *Sagitta*) within intensified potato-based cropping patterns at the Regional Agricultural Research Station (RARS), Burirhat, Rangpur, Bangladesh during the 2017–2018 cropping year. Thirteen cropping systems, including the conventional potato–*boro* rice–T. *aman* rice system, were assessed using a randomized complete block design with three replications. Results showed that incorporation of early-bulking potato genotypes enabled the inclusion of four to five crops per year, substantially increasing system productivity. Potato equivalent yield increased by 43.8–111.5% in the improved patterns compared with the conventional system. The highest land use efficiency (95.61%), whole pattern gross margin (Tk. 538775 ha^−1^), and marginal benefit-cost ratio (8.17) were achieved in the 7 *Alu* – *garden pea* – red amaranth – T. *aus* rice – T. *aman* rice pattern, while the highest potato equivalent yield (95.37 t ha^−1^) was recorded in the 7 *Alu* – cardinal – Mungbean – red amaranth – T. *aman* rice pattern. This study indicates that inclusion of early bulking potato varieties has the potential to serve as effective entry-point crops for intensifying potato-based cropping systems, improving land use efficiency and farm profitability. These findings represent promising outcomes from a single-season trial and highlight the potential of flexible cropping system designs that warrant further multi-year and multi-location validation.

## Introduction

Potato (*Solanum tuberosum* L.), is the world’s third most important food crop after rice and wheat, owing to its high yield potential, short growth duration, and significant nutritional value [1–4]. It plays a vital role in food and nutritional security, particularly in densely populated developing countries [2,5]. In Bangladesh, potato has become both a staple and a cash crop, contributing substantially to farm income and national food supply. National production has increased rapidly over the past two decades; however, rising population pressure, shrinking arable land, and increasing climate variability pose major challenges to sustaining agricultural productivity [6,7].

Agriculture in Bangladesh is dominated by intensive rice-based cropping systems, among which the potato – *boro* rice – T. *aman* rice pattern is widely practiced particularly in the northwestern region [8]. Although this system ensures household food security, it is often associated with low land use efficiency, high irrigation and energy demand, and limited profitability, especially due to the resource intensive nature of *boro* rice cultivation [9,10]. Consequently, diversification and intensification of existing cropping systems through the inclusion of short-duration and high value crops have been identified as key strategies to improve system productivity and economic returns [11,12].

Early-bulking and early-maturing potato genotypes provide an important opportunity to intensify potato-based cropping systems. These genotypes can be harvested within 55–60 days after planting, creating temporal space for additional crops within the same year. Such flexibility allows conventional three crop systems to be redesigned into four- or five-crop sequences, thereby improving annual system productivity and land use efficiency [13,14]. Moreover, the integration of short-duration legumes and vegetables into intensified systems may enhance nutrient cycling, resource-use efficiency, and system resilience under intensive land use [12].

Recent advances in crop breeding, agronomic management, and mechanization have further increased the feasibility of intensified cropping systems. At the same time, climate change manifested through increasing temperature variability and altered rainfall patterns has heightened the need for adaptive and flexible cropping system designs rather than reliance fixed crop sequences or single crop optimization [7,10]. Consequently, system level evaluation of productivity, land use efficiency, and economic performance has become increasingly important for guiding sustainable agricultural intensification.

Despite growing interest in cropping system intensification, limited field-based evidence is available on the performance of early-bulking potato genotypes within redesigned potato-based cropping patterns under Bangladesh conditions. In particular, information on whole-system productivity, economic feasibility, and land use efficiency resulting from the strategic use of early potato as an entry-point crop remains scarce.

Therefore, this study was conducted to evaluate the performance of selected early-bulking potato genotypes (7 *Alu* and *Sagitta*) within existing and improved potato-based cropping patterns. Specifically, the study aimed to assess agronomic performance, system productivity, land use efficiency, and economic returns of intensified cropping systems under field conditions. The findings provide indicative evidence from a single-season trial on the potential of early potato-based system redesign to enhance cropping intensity and farm profitability, while highlighting the need for further multi-year and multi-location validation.

## Materials and methods

### Experimental site and soil characteristics

This experiment was conducted at the Regional Agricultural Research Station (RARS), Burirhat, Rangpur under Agro-Ecological Zone of Tista Meander Floodplain (AEZ 3). The experiment was conducted over a single cropping year (26 September 2017 to 24 September 2018), representing a short-term evaluation of the performance of early bulking potato genotypes within intensified cropping patterns. Experimental plot was a high land having low soil organic matter content (<1.80%), good moisture holding capacity and a pH value of 4.6 to 7.0. The texture of the soil was sandy loam to silty clay loam soil. The initial and final health status before and after the experiment is summarized in Supplementary S1 Table. The experimental site is under subtropical monsoon climate characterized by heavy precipitation during the months of April to September and scanty rainfall during the months of October to March. Highest monthly average temperature was 31°C in April and lowest was recorded on January (15°C). Long time low temperature prevails in winter. Average annual rainfall is 216.9 mm.

### Land preparation

Prior to the *Kharif-II* (mid July – mid November) season of 2017, the land was ploughed and cross ploughed several times and then leveled by rotovator. The weed and other stubbles were removed from the field. Except for the first crop, the plots of the experiment were prepared every time by three spading followed by laddering for keeping the layout intact.

### Experimental design and treatments

The experiment was laid out in a randomized complete block design with three replications. Area of each unit plot was 20 m^2^ (5 m × 4 m). Total 13 potato-based cropping patterns were studied in this study. Treatment includes: Potato–*boro* rice–T. *aman* rice (CP_1_), 7 *Alu*–wheat–mungbean–red amaranth–T. *aman* rice (CP_2_), *Sagitta*–wheat–mungbean–red amaranth–T. *aman* rice (CP_3_), 7 Alu–wheat–T. *aus* rice–T. aman rice (CP_4_), *Sagitta*–wheat–T. *aus* rice–T. *aman* rice (CP_5_), 7 *Alu*–cardinal–mungbean-red amaranth–T. *aman* rice (CP_6_), *Sagitta*–cardinal–mungbean–red amaranth –T. *aman* rice (CP_7_), 7 *Alu*–cardinal/maize–T. *aman* rice (CP_8_), *Sagitta*–cardinal/maize–T. *aman* rice (CP_9_), 7 *Alu*–garden pea–red amaranth–T. *aus* rice–T. *aman* rice (CP_10_), *Sagitta*–garden pea–red amaranth–T. *aus* rice–T. *aman* rice (CP_11_), 7 *Alu*–garden pea–*boro* rice–T. *aman* rice (CP_12_), *Sagitta*–garden pea–*boro* rice–T. *aman* rice (CP_13_). Here, CP_1_ is the existing pattern and rests are improved cropping pattern having early maturing variety (7 *Alu* and *Sagitta*). Two early bulking potato genotypes were used as planting material in six patterns along with one existing pattern. Thus, total number of patterns was thirteen (2 × 6 + 1 = 13). For existing pattern, traditional varieties such as cardinal for potato, BRRI dhan28 for *boro* rice and *Swarna* for T. *aman* rice were used. For improvised patterns, Cardinal is replaced by 7 *Alu* or *Sagitta*, and Swarna was replaced by Binadhan-7. BARI Gom26, BARI Mug-6, BARI Lalshak-1, BRRI dhan48, BARI Motorshuti-3 and BARI BHM-9 were used for wheat, mungbean, red amaranth, T. *aus* rice, garden pea and maize, respectively in the improved patterns.

### Crop husbandry

Early bulking potato seed tubers of 35–45 mm were sown on 6 October, 2017 by using spacing 50 cm × 15 cm and Cardinal seed tuber were sown on 21 November, 2017 by using spacing 60 cm × 25 cm in furrow opened by a small type of hand hoe. In case of second potato seed tuber were sown on 3 December, 2017. Seed tubers were covered with soils by making ridge of about 20 cm height after planting. Maize (*Zea mays* L.) seeds were sown on 7 January, 2018, 35 days after planting of potato in furrow along the inter row spacing of potato to maintain 60 cm × 20 cm distance according to treatment. Wheat (*Triticum aestivum* L.) seeds were sown on 3 December, 2017 by using spacing 20 cm × 20 cm. Mungbean (*Vigna radiata* L. R. Wilczek) seeds were sown on 20 March, 2018 for the cropping pattern CP_2_ and CP_3_. In case of cropping pattern CP_6_ and CP_7_ seeds were sown on 5 March, 2018. The seeds were sown in line by using spacing 30 cm × 5 cm. A light irrigation was applied after sowing to ensure soil moisture for germination. Mungbean residues were incorporated with the soil after one picking of pods. The plot was harrowed and irrigated for decomposition of biomass. Red amaranth (*Amaranthus cruentus* L.) seeds were sown on 10 May, 2018 for the cropping pattern CP_6_ and CP_7_. In case of cropping pattern CP_10_ and CP_11_ seeds were sown on 19 February, 2018. Continuous seeding was followed with maintaining 20 cm line to line distance. Garden pea (*Pisum sativum* L.) seeds were sown on 3 December, 2017 for the cropping pattern of CP_10_, CP_11_, CP_12_ and CP_13_. Seeds were sown in line with the spacing of 30 cm × 15 cm. Weeding, pests’ control and other cultural managements were done as per recommended practice.

For raising rice seedlings, 1 m^2^ plot was prepared by ploughing with harrow, and 32-18-42-18 kg ha^-1^ of N-P-K-S was applied to the plot during final land preparation. For maintaining proper seed rate, 100 g seed of each variety was soaked in water for 24 hours and then kept in bag for 48 hours (72 hours for *boro* rice). The sprouted seeds were broadcast in the seedbed on 20 January, 2018 for *boro* rice, 7 March 2018 for T. *aus* rice, 2 June for T. *aman* rice (Binadhan-7) and 17 June 2018 for T. *aman* rice (Swarna). Seedlings aged 35 days were transplanted in each plot with a spacing of 20 cm × 15 cm.

Fertilization of the crops in the experimental plots were carried out as per their respective recommended doses (Table 1). All crops received half dose of nitrogen fertilizer and full dose of all other fertilizers as basal and the remaining N fertilizer were top dressed after planting or transplanting, but for potato it was top dressed after second earthing up that was 25 days after planting. The rest amount of N for maize were incorporated with soil in rows in two equals splits after irrigation at 25 and 50 days after emergence (DAE) and for wheat it was top dressed at 21days just after first irrigation. The rest amounts of N for *boro* and T. *aman* rice were applied in two equal installments at 20 and 40 days after transplanting. The rest amount of N for T. *aus* was applied at 20 and 35 days after transplanting. Irrigation was done at 28, 40 and 50 days after planting of early potato and at 75 days after planting of maize (after potato harvest) and at 100 days after planting of maize. Three irrigations were applied in wheat at CRI, maximum tillering and early grain filling stages. Six irrigations for T. *aman* rice, ten irrigations for T. *aus* rice and 20 irrigations for *boro* rice were applied during the growing period.

**Table 1:** Variety, applied fertilizer doses and maintained spacing of different crops under studied cropping pattern.

Crops	Variety	Fertilizer N-P-K-S-Zn-B-Mg-Cowdung (kg ha^-1^)	Spacing cm^2^	Plot size (m^2^)
Potato	7 Alu	225-150-250-120-10-100-10-10000	50 × 15	20 m^2^
	Sagitta			
	Cardinal	225-150-250-120-10-100-10-10000	60 × 25	20 m^2^
	Cardinal (Potato- Potato)	135-90-150-72-6-60-6-6000		20 m^2^
*Boro* rice	BRRI dhan28	164-60-104-67-0-0-0-0	20 × 15	20 m^2^
T. *aman* rice	Swarna	100-0-0-15-0-0-0-0		20 m^2^
		Binadhan-7	150-110-50-50-10-0-0-0	
Wheat	BARI Gom-26	220-180-50-120-0-0-0-10000	20 × 20	20 m^2^
Maize	BARI Hybrid Maize −9	250-120-90-120-5-2.5-19-4000	60 × 20	20 m^2^
Garden pea	BARI motorshuti-3	35-85-40-55-0-10-0-0	30 × 5	20 m^2^
Mungbean	BARI Mug-6	50-85-35-0-0-0-0-0	30 × 5	20 m^2^
Red amaranth	BARI Lalshak-1	15-0-0-0-0-0-0-0	Continuous seeding with line 20 cm apart	20 m^2^

### Harvesting, processing and data collection

The traditional potato was harvested after 80−90 days of planting when the plants showed 80−90% of leaf senescence and drying up of the tops which indicated the maturity of plants. Data on tuber yield (t ha^-1^) were recorded at harvest. While, for short duration potato, the crop was harvested after 55 days of planting and both the marketable tuber yield (t ha^-1^) and unmarketable tuber yield (t ha^-1^) was recorded at harvest. After shelling the maize cobs, the grain weight was taken at 14% moisture content and stover yield was recorded by oven drying 10 randomly selected plants from each plot at 70°C for 72 hours. Wheat was harvested at maturity for grain and straw yields and was adjusted at 12% moisture content and expressed in t ha^-1^. Grain and straw were sun dried until moisture content reached at 12%. Grain and straw yields (t ha^-1^) was recorded. When rice plant was in mature stage, it was harvested and yield data (t ha^-1^) were calculated for 14% moisture content. Yield of mungbean, garden pea and red amaranth was also expressed as t ha^-1^.

Productivity of a cropping pattern refers to the annual yield of different crops accommodated into a particular cropping pattern. In this experiment, productivity means the summation of yield of the main crop (Potato) and potato equivalent yield (PEY) of other crops. After harvesting every component yield were recorded and potato equivalent yield (PEY) was calculated using the following equation [15]:


$$\text{PEY}=\;\frac{\text{Yield of component crop }\times \text{ Price of component crop}}{\text{Price of main crop}}\;$$ (1)

Land use efficiency (LUE) was estimated using the following formula [16]:


$$\text{LUE}=\;\frac{\sum {\text{D}}_{\text{c}}}{\text{365}}$$ (2)

Where,

D_c_ = Time span of crops in a sequence.

Marginal benefit cost ratio (MBCR) was calculated following the formula described by Rahman et al. [17]. While calculating the MBCR, only the cost of material and non-material inputs was taken into consideration in the computation of cost and return.


$$\text{MBCR}=\;\frac{\text{Gross return of the improved pattern }-\text{ Gross return of the existing pattern}}{\text{Cost of improved pattern }-\text{ Cost of the existing pattern}}$$ (3)

The costs of production of different cropping patterns set in the experiment were calculated considering only the material and non-material input costs. The gross returns were calculated by multiplying the total production of the cultivated crops ha^-1^ and their unit value in the market. Here unit value was considered as BDT kg^-1^ and crops, output as t ha^-1^ at the same time the gross margin was also calculated by the following formula:


Gross margin = Gross return−Total variable cost of production of respective crop (4)

## Results

### Component yield of the cropping pattern

The yield performance of thirteen potato-based cropping patterns under the study is summarized in the Table 2. The outcome demonstrated that, using Cardinal variety in existing cropping pattern (CP_1_) resulted in highest potato tuber yield (28.72 t ha^−1^) which was planted in 21 November and remained in the field for 95 days. On other patterns using 7 *Alu* and *Sagitta*, 17.70–19.24 t ha^−1^ and 16.45–17.20 t ha^−1^ tuber yield, respectively was found at 55 days after planting (DAP). Production of BRRI dhan28 was highest in existing pattern (5.43 t ha^−1^). Among the improved patterns, CP_12_ showed highest yield (5.31 t ha^−1^) where it was planted after garden pea. Swarna, variety of T. *aman* rice, resulted the highest yield of 4.42 t ha^−1^ in existing the patterns whereas yield of Binadhan-7 ranged from 3.25 to 3.46 t ha^−1^ in the improved patterns. Average wheat yield was found 4.03, 4.21, 4.65 and 4.86 t ha^−1^ from the improved cropping patterns CP_2_, CP_3_, CP_4_ and CP_5_, respectively. Mung bean gave grain yield of 0.80, 0.82, 0.84 and 0.81 t ha^−1^ under the improved cropping patterns of CP_2_, CP_3_, CP_6_ and CP_7_, respectively. Yield of red amaranth ranged from 11.95 to 12.34 t ha^−1^ as a component crop of four patterns investigated. Maize yield was 8.1 and 8.04 t ha^−1^ in CP_8_ and CP_9_, respectively while garden pea produced grain yield of 6.70, 6.60 6.80 and 6.90 t ha^−1^, respectively from CP_10_, CP_11_, CP_12_ and CP_13_. T. *aus* rice yield was 4.52, 4.41, 4.10 and 4.38 t ha^−1^ in improved cropping patterns of CP_4_, CP_5_, CP_10_ and CP_11_, respectively.

**Table 2:** Performance of individual crop and total productivity of existing and improved potato -based cropping patterns studied at RARS, Burirhat, Rangpur during 2017-2018.

Cropping Patterns	Crop	Genotype	Date of planting (DD-MM-YYYY)	Date of harvest (DD-MM-YYYY)	Field duration (days)	Turn Around Period (days)	Individual Yield of patterns crop (t ha^-1^)	Straw/Stover yield t ha^-1^	Individual PEY of the patterns crop (t ha^-1^)
**Existing pattern CP**_**1**_	Potato	Cardinal	21.11.2017	23.2.2018	95	10	28.72	–	28.72
	*Boro* rice	BRRI dhan28	26.2.2018	15.6.2018	110	2	5.43	6.24	8.48
	T. *aman* rice	Swarna	18.7.2018	10.11.2018	116	32	4.42	5.3	7.92
**Improved Pattern CP**_**2**_	Potato	7 Alu	6.10.2017	29.11.2017	55	10	19.24	1.48	49.98
	Wheat	BARI Gom26	3.12.2017	18.3.2018	106	2	4.03	5.24	8.7
	Mungbean	BARI Mug-6	20.3.2018	22.5.2018	64	2	0.8	–	4.4
	Red amaranth	BARI Lalshak-1	25.5.2018	23.6.2018	30	2	10.64	–	11.17
	T. *aman* rice	Binadhan-7	1.7.2018	25.9.2018	87	7	3.88	4.64	6.94
**Improved Pattern CP**_**3**_	Potato	Sagitta	6.10.2017	29.11.2017	55	10	16.6	1.82	43.32
	Wheat	BARI Gom26	3.12.2017	18.3.2018	106	2	4.21	5.17	8.43
	Mungbean	BARI Mug-6	20.3.2018	22.5.2018	64	2	0.82	–	4.51
	Red amaranth	BARI Lalshak-1	25.5.2018	23.6.2018	30	2	10.9	–	11.45
	T. *aman rice*	Binadhan-7	1.7.2018	25.9.2018	87	7	3.92	4.7	6.68
**Improved Pattern CP**_**4**_	Potato	7 Alu	6.10.2017	29.11.2017	55	10	18.54	1.67	48.02
	Wheat	BARI Gom26	3.12.2017	18.3.2018	106	3	4.65	5.08	8.33
	T. *aus* rice	BRRI dhan48	1.4.2018	19.6.2018	80	13	4.52	5.42	6.63
	T. *aman* rice	Binadhan-7	1.7.2018	25.9.2018	87	11	3.3	3.96	5.58
**Improved Pattern CP**_**5**_	Potato	Sagitta	6.10.2017	29.11.2017	55	10	17.02	1.96	44.51
	Wheat	BARI Gom26	3.12.2017	18.3.2018	106	3	4.86	5.83	8.43
	T. *aus* rice	BRRI dhan48	1.4.2018	19.6.2018	80	13	4.41	5.29	6.39
	T. *aman* rice	Binadhan-7	1.7.2018	25.9.2018	87	11	3.25	3.9	5.54
**Improved Pattern CP**_**6**_	Potato	7 Alu	6.10.2017	29.11.2017	55	10	17.7	1.44	45.69
	Potato	Cardinal	3.12.2017	2.3.2018	105	3	25.6	–	25.6
	Mungbean	BARI Mug-6	5.3.2018	7.5.2018	64	2	0.84	–	4.62
	Red amaranth	BARI Lalshak-1	10.05.2018	9.6.2018	30	2	12.01	–	12.81
	T. *aman* rice	Binadhan-7	1.7.2018	25.9.2018	87	21	3.9	4.68	6.65
**Improved Pattern CP**_**7**_	Potato	Sagitta	6.10.2017	29.11.2017	55	10	16.9	1.96	44.21
	Potato	Cardinal	3.12.2017	2.3.2018	105	3	25.54	–	25.54
	Mungbean	BARI Mug-6	5.3.2018	7.5.2018	64	2	0.81	–	4.46
	Red amaranth	BARI Lalshak-1	10.05.2018	9.6.2018	30	2	11.95	–	12.55
	T. *aman* rice	Binadhan-7	1.7.2018	25.9.2018	87	21	3.9	4.68	6.67
**Improved Pattern CP**_**8**_	Potato	7 Alu	6.10.2017	29.11.2017	55	10	17.9	1.82	46.57
	Potato/	Cardinal	3.12.2017	3.3.2018	90	3	24.02	–	24.02
	/Maize	BARI BHM-9	7.1.2018	31.5.2018	145	0	8.1	–	12.15
	T. *aman* rice	Binadhan-7	1.7.2017	25.9.2017	87	30	3.25	4.06	5.54
**Improved Pattern CP**_**9**_	Potato	Sagitta	6.10.2017	29.11.2017	55	10	17	2.2	44.7
	Potato/	Cardinal	3.12.2017	3.3.2018	90	3	24.15	–	24.15
	/Maize	BARI BHM-9	7.1.2018	31.5.2018	145	0	8.04		12.06
	T. *aman* rice	Binadhan-7	1.7.2018	25.9.2018	87	30	3.45	4.26	5.86
**Improved Pattern CP**_**10**_	Potato	7 Alu	6.10.2017	29.11.2017	55	10	18.60	1.86	48.37
	Garden pea	BARI Motorshuti-3	3.12.2017	15.2.2018	75	3	6.7	–	10.05
	Red amaranth	BARI Lalshak-1	19.2.2018	20.3.2018	30	3	12.11	–	12.72
	T. *aus* rice	BRRI dhan48	1.4.2018	19.6.2018	80	11	4.1	4.92	6.34
	T. *aman* rice	Binadhan-7	1.7.2018	25.9.2018	87	11	3.96	4.75	6.75
**Improved Pattern CP**_**11**_ **(Sagitta)**	Potato	Sagitta	6.10.2017	29.11.2017	55	10	16.28	2.1	42.8
	Garden pea	BARI Motorshuti-3	3.12.2017	15.2.2018	75	3	6.6	–	9.9
	Red amaranth	BARI Lalshak-1	19.2.2018	20.3.2018	30	3	12.34	–	12.96
	T. *aus* rice	BRRI dhan48	1.4.2018	19.6.2018	80	11	4.38	5.12	6.85
	T. *aman* rice	Binadhan-7	1.7.2018	25.9.2018	87	11	3.95	4.74	6.93
**Improved Pattern CP**_**12**_	Potato	7 Alu	6.10.2017	29.11.2017	55	10	18.01	1.48	46.51
	Garden pea	BARI Motorshuti-3	3.12.2017	15.2.2018	75	3	6.8	–	10.2
	Boro rice	BRRI dhan28	18.2.2018	6.6.2018	110	2	5.31	6.11	8.29
	T. *aman* rice	Binadhan-7	1.7.2018	25.9.2018	87	24	3.7	4.44	6.3
**Improved Pattern CP**_**13**_	Potato	Sagitta	6.10.2017	29.11.2017	55	10	16.45	2. 01	43.14
	Garden pea	BARI Motorshuti-3	3.12.2017	15.2.2018	75	3	6.9	–	10.35
	T. *aus* rice	BRRI dhan28	1.4.2018	19.6.2018	109	2	5.25	6.08	8.2
	T. *aman* rice	Binadhan-7	1.7.2018	25.9.2018	87	24	3.96	4.75	6.75

PEY = Potato equivalent yield, Potato = 10 Tk. kg^-1^ (Normal market price) Potato = 25 Tk. kg^-1^ (Early market price), Potato = 10 Tk. kg^-1^ (Unmarketable tuber), Wheat = 20 Tk kg^-1^ and Wheat straw = 1Tk. kg^-1^, *Boro* rice = 14 Tk. kg^-1^ and Rice straw = 1.4 Tk. kg^-1,^ T. *aus* rice = 13 Tk. kg^-1^ and rice straw = 1.25 Tk. kg^-1^, T. *aman* rice (Swarna) = Tk. 16.25 kg^-1^ and Rice straw = 1.4 Tk. kg^-1^, T. *Aman* rice (Binadhan-7) = 15 Tk. kg^-1^ and Rice straw = 1.6 Tk. kg^-1^, Garden pea = 14 Tk. kg^-1^, Red amaranth = 10.5Tk kg^-1^, T. *aus* rice = 14 Tk. kg^-1^ and Rice straw = 1.25 Tk. kg^-1^.

### Straw/stover yield

In existing cropping pattern, BRRI dhan28 resulted in highest straw yield of 6.24 t ha^−1^ followed by 6.11 t ha^−1^ in CP_12_ (Table 2). In case of T. *aman* rice, Swarna resulted the highest straw yield of 5.30 t ha^−1^ in the existing pattern whereas straw yield of Binadhan-7 recorded 3.90 to 4.68 t ha^−1^ in the improved patterns. Average wheat straw yield was 5.24, 5.17, 5.08 and 5.83 t ha^−1^ from the improved cropping patterns CP_2_, CP_3_, CP_4_ and CP_5_, respectively (Table 2).

### Crop duration

**Fig 1:**
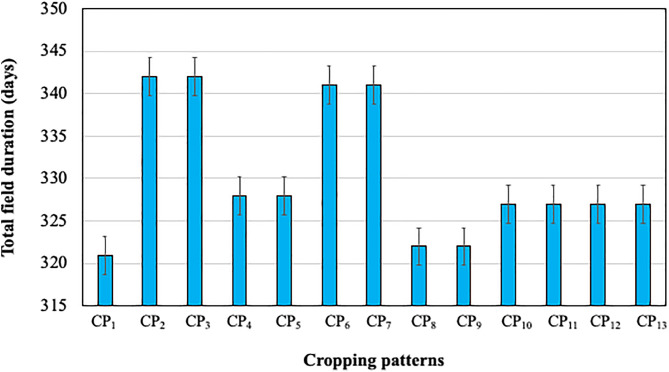
Total field duration of the twelve improved cropping patterns along with existing one during 2017–2018.

Excluding seedling ages of *boro* rice, T. *aus* rice and T. *aman* rice, highest average field duration (342 days) was observed in both CP_2_ and CP_3_ while lowest average field duration (321 days) was observed in the existing one (CP_1_) (Fig 1). It can be concluded that the short duration potato, T. *aus* rice, wheat and leafy vegetable could easily be fitted in the cropping patterns.

### *Potato equivalent yield* (*PEY*)

**Fig 2:**
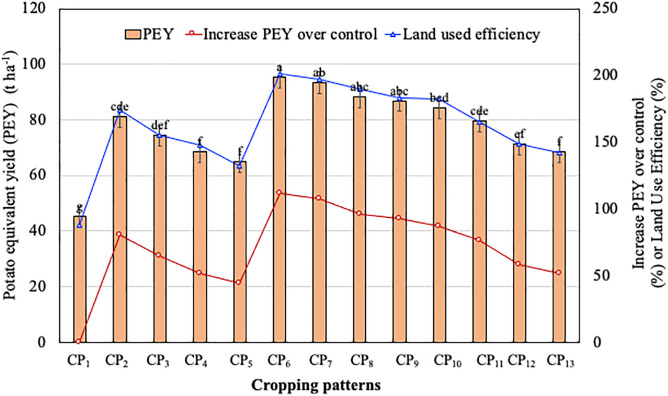
Potato equivalent yield (PEY), percent increase of PEY and land use efficiency of improved cropping patterns over existing one under AEZ-3 during 2017–2018. Potato–*boro* rice–T. *aman* rice (CP_1_), 7 *Alu*–wheat–mungbean–red amaranth–T. *aman* rice (CP_2_), *Sagitta*–wheat–mungbean–red amaranth–T. *aman* rice (CP_3_), 7 Alu–wheat–T. *aus* rice–T. aman rice (CP_4_), *Sagitta*–wheat–T. *aus* rice–T. *aman* rice (CP_5_), 7 *Alu*–cardinal–mungbean-red amaranth–T. *aman* rice (CP_6_), *Sagitta*–cardinal–mungbean–red amaranth –T. *aman* rice (CP_7_), 7 *Alu*–cardinal/maize–T. *aman* rice (CP_8_), *Sagitta*–cardinal/maize–T. *aman* rice (CP_9_), 7 *Alu*–garden pea–red amaranth–T. *aus* rice–T. *aman* rice (CP_10_), *Sagitta*–garden pea–red amaranth–T. *aus* rice–T. *aman* rice (CP_11_), 7 *Alu*–garden pea–*boro* rice–T. *aman* rice (CP_12_), *Sagitta*–garden pea–*boro* rice–T. *aman* rice (CP_13_).

The analysis of variance showed that the highest PEY was recorded in the improved pattern CP_6_ (95.37 t ha^−1^) and the lowest PEY was calculated in existing pattern CP_1_ (45.10 t ha^−1^) (Fig 2). PEY was increased by 43.84 to 111.46% in the improved patterns over traditional farmers’ pattern (CP_1_) (Fig 2).

### *Land use efficiency* (*LUE*)

All the improved cropping patterns showed higher LUE compared to the existing one ranging from 87.75% to 95.61% (Fig 2). Land use efficiency was estimated as the highest of 95.61% in modified cropping pattern and CP_4_, CP_10_, whereas existing cropping pattern (CP_1_) used the land for 87.95% period of the year (Fig 2).

### Cost and return

The whole pattern gross return (GR), Whole pattern gross margin, Benefit over the existing pattern and Marginal benefit cost ratio (MBCR) varied due to variations in yield and cost of production. The highest whole pattern gross margin (538775 Tk. ha^−1^) and Marginal benefit cost ratio (8.17) was obtained from CP_10,_ which was close to CP_6_ (510685 Tk. ha^−1^), CP_2_ (504825 Tk. ha^−1^), CP_7_ (491285 Tk. ha^-1^), CP_10_ (312850 Tk. ha^-1^), and CP_11_ (490875 Tk. ha^−1^) with 1.71, 6.81, 1.65 and 8.05 marginal benefit cost ratio, respectively (Table 3). The lowest whole pattern gross margin (190146 Tk. ha^−1^) was recorded in the existing pattern CP_1_ (Table 3). On the basis of the gross return of a whole pattern (Tk. ha^-1^), the studied patterns can be arranged as CP_6_ > CP_7_ > CP_8_ > CP_9_ > CP_10_ > CP_2_ > CP_11_ > CP_3_ > CP_12_ > CP_4_ > CP_5_ > CP_1_. Considering the total gross margin of a whole pattern (Tk. ha^−1^), the pattern can be arranged as CP_10_ > CP_6_ > CP_2_ > CP_7_ > CP_11_ > CP_3_ > CP_12_ > CP_8_ > CP_9_ > CP_4_ > CP_5_ > CP_1_. Benefit over the existing pattern exhibited the following ranking: CP_10_ > CP_6_ > CP_2_ > CP_7_ > CP_11_ > CP_3_ > CP_12_ > CP_8_ > CP_9_ > CP_4_ > CP_5_ > CP_1_, respectively.

**Table 3:** Cost and return of the existing and improved cropping patterns studied during 2017–2018.

Cropping pattern	Whole pattern gross return (Tk. ha^-1^)	Whole pattern total variable cost (Tk. ha^-1)^	Whole pattern gross margin (Tk. ha^-1^)	Benefit over existing pattern (Tk. ha^-1^)	MBCR (Whole pattern)
CP_1_	451000	260854	190146e	0	
CP_2_	811900	307075	504825ab	314679	6.81
CP_3_	743900	307075	436825bcd	246679	5.34
CP_4_	685600	310290	375310d	185164	3.75
CP_5_	648700	310290	338410d	148264	3.00
CP_6_	953700	443015	510685ab	320539	1.71
CP_7_	934300	443015	491285abc	301139	1.65
CP_8_	882800	480180	402620 cd	212474	0.96
CP_9_	867700	480180	387520d	197374	0.90
CP_10_	842300	303525	538775a	348629	8.17
CP_11_	794400	303525	490875abc	300729	8.05
CP_12_	713000	297464	415536bcd	225390	7.16
CP_13_	684400	297464	386936d	196790	6.38
Level of sig.			**		
LSD			99698		
CV (%)			14.06		

Potato–*boro* rice–T. *aman* rice (CP_1_), 7 *Alu*–wheat–mungbean–red amaranth–T. *aman* rice (CP_2_), *Sagitta*–wheat–mungbean–red amaranth–T. *aman* rice (CP_3_), 7 Alu–wheat–T. *aus* rice–T. aman rice (CP_4_), *Sagitta*–wheat–T. *aus* rice–T. *aman* rice (CP_5_), 7 *Alu*–cardinal–mungbean-red amaranth–T. *aman* rice (CP_6_), *Sagitta*–cardinal–mungbean–red amaranth –T. *aman* rice (CP_7_), 7 *Alu*–cardinal/maize–T. *aman* rice (CP_8_), *Sagitta*–cardinal/maize–T. *aman* rice (CP_9_), 7 *Alu*–garden pea–red amaranth–T. *aus* rice–T. *aman* rice (CP_10_), *Sagitta*–garden pea–red amaranth–T. *aus* rice–T. *aman* rice (CP_11_), 7 *Alu*–garden pea–*boro* rice–T. *aman* rice (CP_12_), *Sagitta*–garden pea–*boro* rice–T. *aman* rice (CP_13_).

## Discussion

The results of this study demonstrate that redesigning existing potato-based cropping systems through the inclusion of early-bulking potato genotypes substantially enhanced system productivity, land use efficiency, and economic returns compared with the conventional potato–*boro* rice–T. *aman* rice pattern. Although the existing pattern produced higher yields of individual long-duration crops such as potato and *boro* rice, the improved cropping patterns achieved superior annual system performance by accommodating additional crops within the same land area and time frame. This confirms that cropping system productivity should be evaluated at the system level rather than on the basis of single-crop yield alone, as emphasized in earlier studies on cropping system intensification [10,11].

The comparatively lower tuber yield of early-bulking potato genotypes (7 *Alu* and *Sagitta*) harvested at 55 days after planting (DAP) was expected, as yield is strongly influenced by both varietal characteristics and crop duration. Similar yield reductions associated with early harvest have been reported in other studies; however, early harvest creates a critical temporal advantage that allows the inclusion of additional crops within the annual cycle [13,14]. In the present study, this temporal flexibility enabled the incorporation of short-duration crops such as mungbean, garden pea, and leafy vegetables, which substantially increased potato equivalent yield (PEY) by 43.8–111.5% over the existing system. These findings are consistent with previous reports that intensification and diversification of rice – and potato – based systems significantly increase system equivalent yield due to higher aggregate production and favorable market prices of component crops [9,10,18].

Land use efficiency was markedly higher in the improved cropping patterns, reaching up to 95.6%. compared with 87.9% in the conventional system. This increase reflects more effective utilization of available land throughout the year by minimizing fallow periods and optimizing crop sequencing. Similar improvements in land use efficiency through the inclusion of short-duration crops have been reported in earlier studies on intensive cropping systems in South Asia [11,16]. The higher field occupation in improved patterns indicates that early potato genotypes can function as effective entry-point crops for system intensification without extending the total annual cropping duration beyond manageable limits.

Economic analysis further supported the agronomic advantages of the improved cropping patterns. The substantially higher whole-pattern gross margin and marginal benefit-cost ratio observed in patterns such as 7 *Alu* – garden pea – red amaranth – T. *aus* rice – T. *aman* rice (CP_10_) highlight the economic feasibility of intensification. These gains were driven by a combination of increased cropping intensity, higher cumulative yields, and inclusion of high value short duration crops. Similar economic benefits of intensified and diversified cropping systems have been documented in Bangladesh and neighboring regions, where replacing fallow or low-return crops with legumes and vegetables significantly increased farm profitability [10,13,19].

From sustainability perspectives, intensified cropping systems raise concerns regarding long term soil fertility and resource use. However, the inclusion of leguminous crops such as mungbean and garden pea in several improved patterns may contribute to partial nitrogen replenishment and improved nutrient cycling, thereby supporting system resilience under intensive land use. Previous studies have reported positive effects of legumes on system productivity and sustainability in rice – and potato – based cropping systems [12,14]. While the present study focused primarily on agronomic and economic performance, the observed improvements in productivity and profitability suggest that well-designed intensive systems can be both productive and potentially sustainable when appropriate crop combinations are used.

It is important to note that the findings of the study are based on a single cropping year (2017–2018), and inter annual climatic variability may influence crop performance and system outcomes [20]. Climate variability has been shown to significantly affect potato productivity and cropping system performance in Bangladesh [7]. Therefore, the varietal performance and system advantages observed in this study should be considered indicative rather than definitive. Multi-year and multi-location evaluations are required to confirm the stability, adaptability, and long-term sustainability of the proposed cropping patterns under varying climatic and management conditions.

Although the experimental data were generated during 2017–2018 cropping season, the findings remain relevant in the current context as they demonstrate a flexible cropping system framework rather than reliance on specific cultivars. Since 2018, advances in agricultural technology, the release of newer short-duration and stress-tolerant varieties, and increasing adoption of mechanization may further enhance the feasibility and profitability of such intensified systems. However, ongoing climate change, including increased temperature variability and altered rainfall patterns, may also influence crop performance and economic outcomes. Therefore, the proposed cropping patterns should be viewed as adaptable models that can be updated with newly released varieties and site-specific management practices.

Finally, the results align well with earlier research demonstration that strategic intensification of existing cropping systems through short duration crops can substantially enhance land productivity, profitability, and resource use efficiency [10–12]. The present study extends this knowledge by highlighting the role of early-bulking potato genotypes as a practical entry point for redesigning potato-based cropping systems in Bangladesh.

## Conclusions

This study demonstrated that intensification of existing potato – based cropping systems through the inclusion of early-bulking potato genotypes can substantially enhance system productivity, land use efficiency, and economic returns compared with the conventional potato – *boro* rice – T. *aman* rice pattern. Although early-bulking genotypes produced lower individual potato yields due to shorter duration, their early harvest created temporal space for the inclusion of additional short-duration crops, resulting in higher annual system productivity and profitability. Among the evaluated systems, the 7 *Alu –* garden pea – red amaranth – T*. aus* rice *–* T. *aman* rice pattern showed the highest economic benefit, while 7 Alu – cardinal – mungbean – red amaranth – T. *aman* rice appeared particularly suitable for regions practicing double potato cultivation. Improved cropping patterns also achieved higher land use efficiency by reducing fallow periods and optimizing crop sequencing. Moreover, inclusion of early potato varieties have the potential to harvest four to five crops per year from a piece of land that increases both land use efficiency and potato equivalent yield. Consequently, the improved cropping intensity will enhance food security and nutritional security of the nation. The proposed cropping patterns therefore provide a practical framework that can be adapted using improved varieties and contemporary management practices to enhance productivity under evolving climatic and technological conditions.

## Study limitations and future research

As the study was conducted over a single cropping year, seasonal climatic variability and management conditions may have influenced system productivity. Therefore, caution is required when extrapolating these findings across years and agro-ecological conditions, and multi-year, multi-location studies are recommended to validate the performance and sustainability of the intensified cropping patterns.

**Highlights**

- Early-bulking potato genotypes enabled the inclusion of four to five crops per year within existing potato-based cropping systems.
- Intensified cropping patterns significantly improved land use efficiency, system productivity, and whole-pattern profitability compared with the conventional system.
- Inclusion of short-duration legumes and vegetables enhanced system resilience and economic returns, demonstrating the potential for sustainable cropping system intensification.

## Supporting information

S1 Table Pre (initial) and post-experiment soil fertility across cropping patterns. Here, Potato–*boro* rice–T. *aman* rice (CP_1_), 7 *Alu*–wheat–mungbean–red amaranth–T. *aman* rice (CP_2_), *Sagitta*–wheat–mungbean–red amaranth–T. *aman* rice (CP_3_), 7 Alu–wheat–T. *aus* rice–T. aman rice (CP_4_), *Sagitta*–wheat–T. *aus* rice–T. *aman* rice (CP_5_), 7 *Alu*–cardinal–mungbean-red amaranth–T. *aman* rice (CP_6_), *Sagitta*–cardinal–mungbean–red amaranth –T. *aman* rice (CP_7_), 7 *Alu*–cardinal/maize–T. *aman* rice (CP_8_), *Sagitta*–cardinal/maize–T. *aman* rice (CP_9_), 7 *Alu*–garden pea–red amaranth–T. *aus* rice–T. *aman* rice (CP_10_), *Sagitta*–garden pea–red amaranth–T. *aus* rice–T. *aman* rice (CP_11_), 7 *Alu*–garden pea–*boro* rice–T. *aman* rice (CP_12_), *Sagitta*–garden pea–*boro* rice–T. *aman* rice (CP_13_).
(DOCX)
