# Supplementary material for: Enhancing cropping intensity and system productivity through early-bulking potato genotypes in potato-based cropping systems
Source: PLoS One. 2026 Mar 17;21(3):e0344679. doi: 10.1371/journal.pone.0344679 (PMC12994817; doi:10.1371/journal.pone.0344679)
Supplement: S1 Table — Here, Potato–boro rice–T. aman rice (CP1), 7 Alu–wheat–mungbean–red amaranth–T. aman rice (CP2), Sagitta–wheat–mungbean–red amaranth–T. aman rice (CP3), 7 Alu–wheat–T. aus rice–T. aman rice (CP4), Sagitta–wheat–T. aus rice–T. aman rice (CP5), 7 Alu–cardinal–mungbean-red amaranth–T. aman rice (CP6), Sagitta–cardinal–mungbean–red amaranth –T. aman rice (CP7), 7 Alu–cardinal/maize–T. aman rice (CP8), Sagitta–cardinal/maize–T. aman rice (CP9), 7 Alu–garden pea–red amaranth–T. aus rice–T. aman rice (CP10), Sagitta–garden pea–red amaranth–T. aus rice–T. aman rice (CP11), 7 Alu–garden pea–boro rice–T. aman rice (CP12), Sagitta–garden pea–boro rice–T. aman rice (CP13). (DOCX) [file pone.0344679.s001.docx]

**Supporting Information**

**S1 Table.** Pre (initial) and post-experiment soil fertility across cropping patterns

|  | Cropping  Patterns | pH | OM (%) | N (%) | K | Mg | P | S | Zn | B |
| --- | --- | --- | --- | --- | --- | --- | --- | --- | --- | --- |
|  |  |  |  |  | meq / 100g soil | | Micro gram / g soil | | | |
| Pre-sowing soil (Initial) |  | 5.6 | 1.8 | 0.1 | 0.3 | 0.5 | 48.6 | 16.7 | 0.9 | 0.2 |
| Post-experiment | CP_1_ | 6.1 | 1.85 | 0.08 | 0.13 | 0.45 | 20.36 | 13.44 | 0.46 | 0.12 |
|  | CP_2_ | 5.9 | 1.92 | 0.08 | 0.19 | 0.55 | 45.17 | 17.18 | 0.68 | 0.19 |
|  | CP_3_ | 6.0 | 2.06 | 0.09 | 0.11 | 0.50 | 26.51 | 17.83 | 0.84 | 0.19 |
|  | CP_4_ | 5.7 | 1.89 | 0.07 | 0.17 | 0.54 | 41.20 | 16.53 | 0.70 | 0.17 |
|  | CP_5_ | 5.7 | 1.89 | 0.07 | 0.17 | 0.54 | 41.20 | 16.53 | 0.70 | 0.17 |
|  | CP_6_ | 6.0 | 1.78 | 0.09 | 0.12 | 0.39 | 21.87 | 15.25 | 0.38 | 0.13 |
|  | CP_7_ | 6.1 | 1.88 | 0.12 | 0.18 | 0.53 | 21.75 | 17.39 | 0.80 | 0.18 |
|  | CP_8_ | 5.9 | 1.82 | 0.10 | 0.14 | 0.47 | 30.24 | 14.45 | 0.83 | 0.13 |
|  | CP_9_ | 5.9 | 1.58 | 0.09 | 0.12 | 0.48 | 28.53 | 14.26 | 0.82 | 0.11 |
|  | CP_10_ | 5.7 | 1.89 | 0.09 | 0.16 | 0.58 | 41.10 | 16.11 | 0.72 | 0.16 |
|  | CP_11_ | 5.8 | 1.89 | 0.08 | 0.16 | 0.43 | 39.49 | 17.07 | 0.70 | 0.16 |
|  | CP_12_ | 6.0 | 1.92 | 0.08 | 0.19 | 0.55 | 45.17 | 17.18 | 0.68 | 0.19 |
|  | CP_13_ | 6.0 | 1.90 | 0.08 | 0.20 | 0.57 | 45.26 | 17.01 | 0.66 | 0.18 |

Here, Potato–*boro* rice–T. *aman* rice (CP_1_), 7 *Alu*–wheat–mungbean–red amaranth–T. *aman* rice (CP_2_), *Sagitta*–wheat–mungbean–red amaranth–T. *aman* rice (CP_3_), 7 Alu–wheat–T. *aus* rice–T. aman rice (CP_4_), *Sagitta*–wheat–T. *aus* rice–T. *aman* rice (CP_5_), 7 *Alu*–cardinal–mungbean-red amaranth–T. *aman* rice (CP_6_), *Sagitta*–cardinal–mungbean–red amaranth –T. *aman* rice (CP_7_), 7 *Alu*–cardinal /maize–T. *aman* rice (CP_8_), *Sagitta*–cardinal /maize–T. *aman* rice (CP_9_), 7 *Alu*–garden pea–red amaranth–T. *aus* rice–T. *aman* rice (CP_10_), *Sagitta*–garden pea–red amaranth–T. *aus* rice–T. *aman* rice (CP_11_), 7 *Alu*–garden pea–*boro* rice–T. *aman* rice (CP_12_), *Sagitta*–garden pea–*boro* rice–T. *aman* rice (CP_13_).
